# The hydrocarbon-degrading marine bacterium Cobetia sp. strain MM1IDA2H-1 produces a biosurfactant that interferes with quorum sensing of fish pathogens by signal hijacking

**DOI:** 10.1111/1751-7915.12016

**Published:** 2013-01-02

**Authors:** C Ibacache-Quiroga, J Ojeda, G Espinoza-Vergara, P Olivero, M Cuellar, M A Dinamarca

**Affiliations:** 1 Laboratorio de Biotecnología MicrobianaUniversidad de Valparaíso; 2 Departamento de Ciencias Químicas y Recursos Naturales, Facultad de Farmacia, Universidad de ValparaísoGran Bretaña 1093, 2360102, Valparaíso, Chile; 3 Centro de Investigaciones Biomédicas, Facultad de Medicina, Universidad de ValparaísoHontaneda 2653, 2341369, Valparaíso, Chile

## Abstract

Biosurfactants are produced by hydrocarbon-degrading marine bacteria in response to the presence of water-insoluble hydrocarbons. This is believed to facilitate the uptake of hydrocarbons by bacteria. However, these diffusible amphiphilic surface-active molecules are involved in several other biological functions such as microbial competition and intra-or inter-species communication. We report the isolation and characterization of a marine bacterial strain identified as *Cobetia* sp. MM1IDA2H-1, which can grow using the sulfur-containing heterocyclic aromatic hydrocarbon dibenzothiophene (DBT). As with DBT, when the isolated strain is grown in the presence of a microbial competitor, it produces a biosurfactant. Because the obtained biosurfactant was formed by hydroxy fatty acids and extracellular lipidic structures were observed during bacterial growth, we investigated whether the biosurfactant at its critical micelle concentration can interfere with bacterial communication systems such as quorum sensing. We focused on *Aeromonas salmonicida* subsp. *salmonicida*, a fish pathogen whose virulence relies on quorum sensing signals. Using biosensors for quorum sensing based on *Chromobacterium violaceum* and *Vibrio anguillarum*, we showed that when the purified biosurfactant was mixed with *N*-acyl homoserine lactones produced by *A. salmonicida*, quorum sensing was inhibited, although bacterial growth was not affected. In addition, the transcriptional activities of *A. salmonicida* virulence genes that are controlled by quorum sensing were repressed by both the purified biosurfactant and the growth in the presence of *Cobetia* sp. MM1IDA2H-1. We propose that the biosurfactant, or the lipid structures interact with the *N*-acyl homoserine lactones, inhibiting their function. This could be used as a strategy to interfere with the quorum sensing systems of bacterial fish pathogens, which represents an attractive alternative to classical antimicrobial therapies in fish aquaculture.

## Introduction

Biosurfactants are cellular structures or molecules formed by variable hydrophilic moieties (ester or alcohol group of neutral lipids; carboxylate group of fatty acids or amino acids; phosphate group of phospholipids; and the carbohydrates of glycolipids) and a more constant hydrophobic moiety (hydrocarbon length-variable chains of fatty acids). In general, the synthesis and assembly of hydrophilic and hydrophobic moieties occur through specific biosynthetic pathways that, depending on the microorganism, produce a variety of surface-active glycolipids, lipopeptides, glycolipopeptides, phospholipids, acylated serine-lactones and hydroxy fatty acids with a wide diversity of biological functions (Das *et al*., 2008a).

In marine ecosystems, diffusible amphiphilic surface-active molecules are involved in: (i) microbial competition, when they exhibit antimicrobial properties (Mukherjee *et al*., 2009), (ii) communication (intra or inter-species), when they act as diffusible signals in quorum sensing (Mohamed *et al*., 2008), (iii) nutrition, when their amphiphilic characteristics favour the accession and uptake of complex water-insoluble substrates (Olivera *et al*., 2009), and (iv) survival by binding and sequestering toxic compounds (Gnanamani *et al*., 2010). Given the above, biosurfactants of marine origin have interesting biotechnological applications (Satpute *et al*., 2010). In addition, the production of biosurfactants by industrial fermentation with marine microorganisms is attractive because it is a selective bioprocess that reduces the use of potable water.

Hydrocarbon-degrading marine bacteria (HDMB), which produce amphiphilic compounds in response to the presence of hydrophobic (aromatic or aliphatic) hydrocarbons (Das *et al*., 2008b), represent interesting sources of biosurfactants. HDMB are a diverse group of microorganisms adapted to different marine ecosystems grouped into: (i) nutritionally versatile hydrocarbon-degrading bacteria and (ii) obligated oil-degrading bacteria (Yakimov *et al*., 2007). Despite this (continuously increasing) biodiversity, the production, characterization and applications of biosurfactants have been studied in a few genera of HDMB.

The study of applied uses of biosurfactants produced by HDMB has been focused on the recovery of polluted environments. Nevertheless, it is known that biosurfactants with antimicrobial activity are produced in response to the presence of competitors, which explains their ecological role and suggests potential applications for the control of infectious diseases (Dusane *et al*., 2011). The antimicrobial potential of HDMB-produced biosurfactants has not been extensively studied. Nevertheless, the ubiquity and dominance displayed by these highly specialized microorganisms (Kasai *et al*., 2002; Golyshin *et al*., 2003) must involve, in addition to their metabolic specificity, other capabilities to exclude competitors.

We have focused on HDMB as sources of biosurfactants involved in microbial competition and their potential use as alternatives to classical antimicrobial therapy. In this work, the strain *Cobetia* sp. MM1IDA2H-1 was isolated and studied as a source of surface-active compounds that interact with the quorum sensing systems of bacterial fish pathogens.

## Results

### Isolation and characterization of a biosurfactant-producing marine bacteria

**Figure 1:**
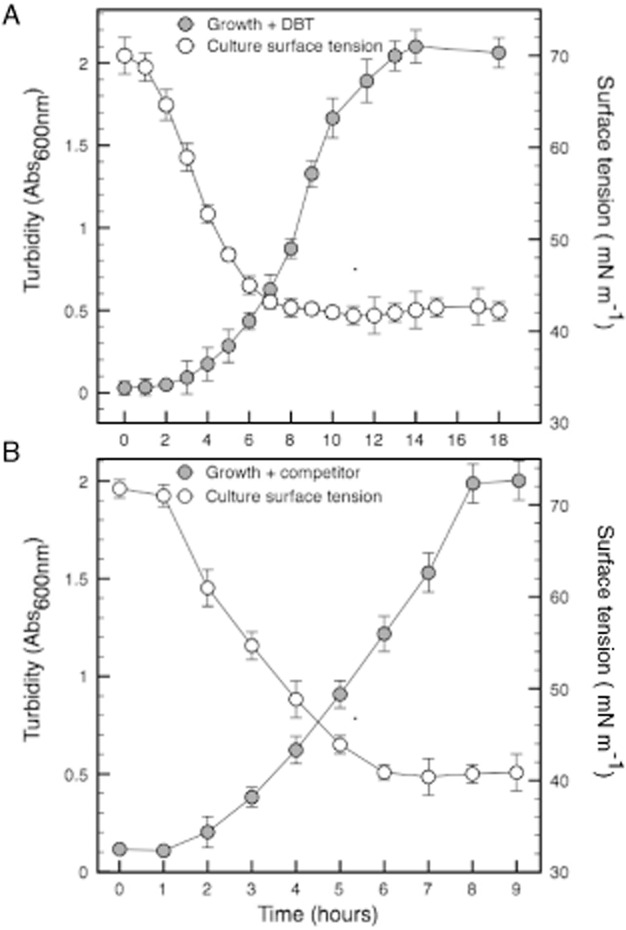
Biosurfactant production by *Cobetia* sp. strain MM1IDA2H-1 in presence of DBT or microbial competitor. The production of surface-active compounds was evaluated by surface tension measures on cell-free supernatants samples obtained during growth in: (A) minimal media containing DBT as the sole carbon source and; (B) minimal media supplemented with succinate 30 mM and containing inactivated cells of the competitor *A. salmonicida*. Error bars indicate standard deviation (*n* = 3).

We isolated the strain MM1IDA2H-1 by selective enrichment using a culture media with the sulfured hydrocarbon dibenzothiophene (DBT) as the sole carbon source and inoculated with seawater samples. We screened 30 isolates as potential sources of surface-active compounds and selected the strain MM1IDA2H-1 because during its growth with DBT, the culture medium surface tension decreased from 70.0 mN m^−1^ to 41.0 mN m^−1^ (Fig. 1A). Likewise, the supernatant obtained formed a stable emulsion with hexadecane for 24 h at 25°C (Table 1). The selected strain was a straight Gram-negative rod that can use citrate, succinate, Tween 40, Tween 80, succinic acid mono-methyl-ester and pyruvic acid methyl-ester as carbon sources. The range of growth in NaCl was from 1% to 18% (w/v), with optimal growth of 8% (w/v) (Supplementary Table S1). Additionally, no growth was achieved in the absence of sodium, confirming that the isolated is a moderately halophilic bacterium, indigenous from the marine ecosystem. Biochemical characterization indicated that the strain MM1IDA2H-1 was positive for oxidase and negative for lysine decarboxylase and nitrate reduction. According to the minimal inhibitory concentration (MIC) evaluated for selected antibiotics, the strain MM1IDA2H-1 was sensitive to amoxicillin, ampicillin, chloramphenicol, gentamicin, kanamycin, rifampycin and streptomycin; and resistant to erythromycin, nalidixic acid, polymyxin B and sulfamide (Supplementary Table S2). The 16S-RNA gene sequence showed 100% similarity to the 16S-RNA of *Cobetia marina* DSM 4741 (Arahal *et al*., 2002). Unlike the strain MM1IDA2H-1, the reference *C. marina* strain DSM 4741 grows in the absence of sodium and is negative for oxidase, β-galactosidase and hydrolysis of Tween 80 (Supplementary Table S1). The metabolism of DBT was confirmed by amplifying a conserved region of the *dszA* gene and detecting the metabolic intermediate 2-hydroxybiphenyl (HBP) in the culture supernatant (Supplementary Table S1). The *dszA* gene encodes a monooxygenase involved in DBT biodesulfurization. The analysis of the sequence obtained from PCR amplification showed 99% similarity to the canonical *dszA* gene of *Rhodococcus erythropolis* strain IGTS8 (GenBank 51949871). It has been established that the *dszA* sequence is highly conserved among bacterial strains that desulfurize DBT (Duarte *et al*., 2001). This gene encodes for a mono-oxygenase belonging to the 4S pathway for the non-destructive desulfurization of DBT, which converts 5,5-dioxide dibenzothiophene (DBTO_2_) to 2-(2′-hydroxyphenyl) benzene sulfinate (HBPSi) (Oldfield *et al*., 1997). However, this mono-oxygenase is a flexible enzyme that also produces 2-hydroxybiphenyl, which explains the presence of this intermediate in supernatants obtained from cultures of the *Cobetia* sp. strain MM1IDA2H-1. The isolated HDMB was denominated *Cobetia* sp. strain MM1IDA2H-1.

**Table 1:** Characterization of biosurfactant produced by *Cobetia* sp. strain MM1IDA2H-1

*Physical*		
Aspect	Yellowish powder	
EI_24_ (%)	44.0 (± 3)	
CMC (mg l^−1^)	80.0 (± 1)	
*Chemical*		
GC-M	Molecule	
	Octadecanoic acid (C_18_H_36_O_2_)	
	(9Z)-Octadec-9-enoic acid (C_18_H_34_O_2_)	
	Hexadec-9-enoic acid (C_16_H_30_O_2_)	
	Hexadecanoic acid (C_16_H_30_O_2_)	
FT-IR	Functional group	Signal
	-CH_3_	2851 cm^−1^
	-CH_2_	2851 cm^−1^
	-C=O	1721 cm^−1^
	-OH	3438 cm^−1^
	NH_1_, NH_2_	Not detected
	COC	Not detected
^1^H-RMN		
	CH_3_	δ 0.88 ppm
	CH_2_	δ 1.20 ppm
	CH_2_-C=O	δ 2.53 ppm
	CH-OH	δ 5.26 ppm
^13^C-NMR		
	C=O	δ 169.2 ppm
	C-2	δ 40.80 ppm
	C-3	δ 67.60 ppm

### *Cobetia* sp. strain MM1IDA2H-1 produces biosurfactant when grown in presence of DBT or a microbial competitor

The strain MM1IDA2H-1 grew with a doubling time of 2 h using DBT as the sole carbon and energy source, with turbidity close to 2.1 (Abs_600 nm_) after 14 h of incubation at 30°C (Fig. 1A). After 8 h of incubation, the surface tension of cell-free supernatant samples obtained from the cultures decreased significantly from 70.0 (± 2.0) mN m^−1^ to 41.0 (± 1.0) mN m^−1^ (Fig. 1A). The reduction in the surface tension stabilized at the exponential phase of growth (8 h of incubation). The cell-free supernatant obtained from this growth phase formed stable emulsions with hexadecane with EI_24_ = 44.0 ± 0.3 (Table 1). In order to determine whether *Cobetia* sp. strain MM1IDA2H-1 produces surfactants in response to ecological stimuli such as the presence of microbial competitors, surface tension of cell-free supernatant obtained from cultures of the strain MM1IDA2H-1 growing in the presence of *Aeromonas salmonicida* cells was measured. Similarly with growth using DBT, the surface tension decreased when grown was in the presence of *A. salmonicida*. Under this condition, after 7 h of incubation the surface tension of filtered supernatant samples decreased significantly from 71.0 (± 1.0) mN m^−1^ to 40.0 (± 2.0) mN m^−1^ (Fig. 1B). The surface tension reduction stabilized at the exponential growth phase (7 h of incubation).

### *Cobetia* sp. strain MM1IDA2H-1 produces external lipid structures when grown with DBT as the sole carbon source

**Figure 2:**
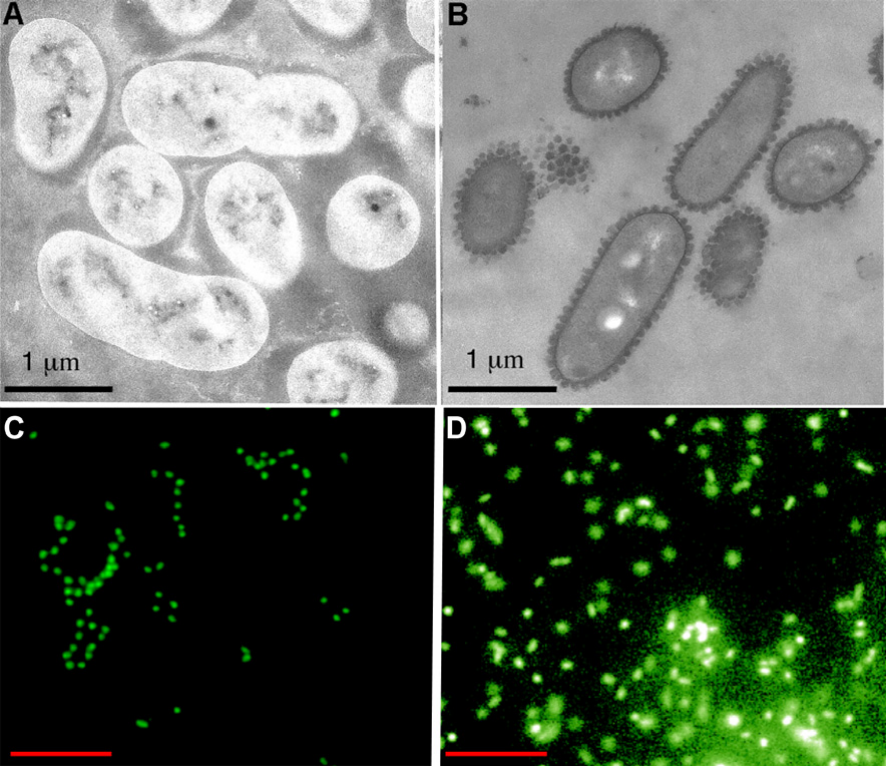
Microscopy of diffusible lipid structures produced by *Cobetia* sp. strain MM1IDA2H-1. Panels (A) and (C) are respectively TEM and epifluorescence images of cells growing on Bushnell-Hass supplemented with succinate 30 mM. Panels (B) and (D) are respectively TEM and epifluorescence images of cells growing with DBT as the only carbon source. For epifluorescence microscopy *Cobetia* sp. strain MM1IDA2H-1 cells were stained with BODIPY 505/515 and SYTO 9 to detect respectively lipidic structures and (nucleic acid) cells (red bar = 10 μm).

Transmission electron microscopy (TEM) revealed the presence of outer cell structures when *Cobetia* sp. strain MM1IDA2H-1 was grown with DBT as the sole carbon source (Fig. 2B). These structures were not observed when succinate was used as the carbon source (Fig. 2A). BODIPY 505/515, a lipophilic fluorescent dye, was used to study the chemical nature of the observed structures (Fig. 2C and D). When cells growing with DBT were stained with BODIPY 505/515 and SYTO 9, green-fluorescence corresponding to lipid extracellular structures and cells was visualized respectively by epifluorescence microscopy (EFM) (Fig. 2D). When succinate was used as the carbon source only green-fluorescence corresponding to nucleic acids of bacterial cells was observed (Fig. 2C). BODIPY presents the problem of producing background fluorescence. However, since the structures observed with this staining technique were only observed when bacterial growth was using DBT and by TEM, we exclude the effect of a technical artefact due to unspecific fluorescence. Considering the selective partitioning of BODIPY 505/515, it is proposed that the observed structures have been formed by lipids.

### Characterization of the biosurfactant produced by *Cobetia* sp. strain MM1IDA2H-1

**Figure 3:**
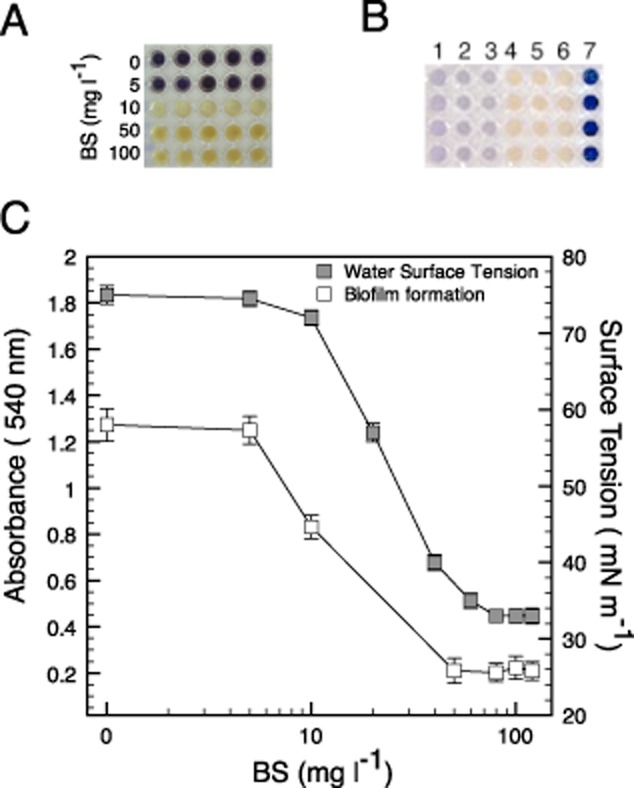
Inhibition of quorum-sensing-dependent phenotypes by the biosurfactant produced using the *Cobetia* sp. strain MM1IDA2H-1.A. Purple phenotype response of *Chromobacterium violaceum* ATCC 12472 to different concentrations (mg ml^−1^) of biosurfactant (BS) produced by the *Cobetia* sp. strain MM1IDA2H-1. The production of the purple pigment violacein is under quorum sensing control in *C. violaceum*, therefore, the loss of this phenotype in the presence of BS was associated with the inhibition of quorum sensing. At the evaluated concentrations no effect on growth was observed.B. Interaction of quorum sensing signals with the biosurfactant. HSLs enriched cell-free supernatants of *A. salmonicida* were mixed with the biosurfactant and used to induce the quorum sensing pigmented phenotype in the HSL not producer strain CV026. 1: *C. violaceum*; 2: CV026 exposed to *C. violaceum* cell-free supernatant; 3: CV026 exposed to *A. salmonicida* cell-free supernatant; 4: strain CV026 unexposed; 5: CV026 exposed to *A. salmonicida* cell-free supernatant mixed with biosurfactant; 6: CV026 exposed to *C. violaceum* cell-free supernatant mixed with biosurfactant; 7: *A. tumefaciens* exposed to *A. salmonicida* cell-free supernatant.C. Surface tension (ST) of water at different concentrations (mg l^−1^ in logarithmic scale) of the BS, and *V. anguillarum* biofilm formation at different concentrations (mg l^−1^ in logarithmic scale). The CMC was established when reduction of ST was stabilized without changes (at 80 mg l^−1^). Values for ST represent the average of three independent assays.

When the supernatant was precipitated with chilled acetone and extracted with solvents, a yellowish powder was obtained that reduced the water surface tension to 33 (± 0.8) mN m^−1^ with a CMC of 80 mg l^−1^ (Table 1, Fig. 3C). The chemical characterization of the biosurfactant is shown in Table 1. The FT-IR spectra showed signals at 2851 cm^−1^, 1721 cm^−1^ and 3438 cm^−1^ associated respectively with: a hydrocarbon chain (-CH_3_ and -CH_2_), a carbonyl group (-C=O) and a hydroxy group (-OH). No bands were detected by this technique related to NH, NH_2_ or COC groups, corresponding to peptides or saccharides respectively. Analysis of ^1^H-RMN of the obtained biosurfactant showed the presence of signals at δ 0.88 ppm (t, CH_3_), δ 1.2 ppm (br.s, CH_2_) and at δ 2.53 ppm (ddd, CH_2_-C=O) (Supplementary Fig. S1). These signals and the FT-IR confirm the presence of aliphatic chains. Additionally, hydroxylation of the hydrocarbon chain was observed in the C-3 position due to the presence of a signal at δ 5.26 ppm (q, CH-OH). In the ^13^C-NMR spectrum signals at δ 169,2 ppm (C=O), δ 40.8 ppm (C-2) and δ 67.6 ppm (C-3) were detected (Supplementary Fig. S2). Using the Wiley 138 library to compare the mass spectrums obtained from the biosurfactant, we found the following molecules to have greater similarity (according to the maximal statistical probability of the match): octadecanoic acid (C_18_H_36_O_2_); octadec-9-enoic acid (C_18_H_34_O_2_); hexadecanoic acid (C_16_H_32_O_2_) and hexadecenoic acid (C_16_H_30_O_2_) (Supplementary Fig. S3). It was proposed that the biosurfactant produced by *Cobetia* sp. strain MM1IDA2H-1 was a mix of 3-hydroxy fatty acids. Because it has been recently described that certain fatty acids can act as signals involved in interspecies cell-to-cell communication, the effect of the biosurfactant produced by *Cobetia* sp. strain MM1IDA2H-1 was evaluated for its potential role in quorum-sensing.

### The biosurfactant produced by *Cobetia* sp. strain MM1IDA2H-1 inhibits cell-to-cell communication based on quorum sensing

The surface-active product was tested for its activity against quorum sensing on *Chromobacterium violaceum* ATCC 12472 exposed to 5, 10, 50 and 100 mg l^−1^ of the biosurfactant (Fig. 3A). *Chromobacterium violaceum* production of the purple pigment violacein is under quorum sensing control. Therefore, the loss of this phenotype is associated with the inhibition of quorum sensing. The minimum concentration of biosurfactant to inhibit quorum sensing, measured by the loss of purple phenotype, was 10 mg l^−1^ (Fig. 3A). At these concentrations, no inhibition of *C. violaceum* growth was observed (Abs_600 nm_ = 1.5).

To determine possible interactions between the surfactant and chemical signals involved in quorum sensing, the strain *C. violaceum* CV026 and a cell-free supernatant obtained from a culture of *A. salmonicida* were used as a biosensor and a source of acyl-HSLs respectively. CV026 is a mutant strain of *C. violaceum* that cannot produce HSLs but produces violacein when exposed to exogenous HSLs with acyl chains ranging from C_4_ to C_6_. When the cell-free supernatant of *A. salmonicida* was used as a source of C_4_-C_6_ HSLs, we observed the induction of violacein production (Fig. 3B, Table 2). Nevertheless, when CV026 was exposed to the acyl-HSLs enriched cell-free supernatant previously mixed with the biosurfactant, no pigmented phenotype was formed (Fig. 3B, Table 2). The presence of HSL signals in the cell-free supernatants of *A. salmonicida* was corroborated using *Agrobacterium tumefaciens* NTL4 as a biosensor. This strain harbouring the pZLR4 plasmid (with gentamicin and carbenicillin resistances) including a *traG::lacZ* fusion and *traR*. When strain NTL4(pZLR4) is exposed to HSL, it will diffuse into the cell and activate the TraR protein. Transcription of the *traG::lacZ* fusion is then activated by TraR. Thus, LacZ (beta-galactosidase) activity was used as a reporter of *traG* transcription, and hence an indicator of the presence of HSL. A blue colour indicating X-gal hydrolysis was recorded as positive for presence of HSL in cell-free supernatant of *A. salmonicida* (Fig. 3B column 7).

**Table 2:** Induction of AHLs depending phenotypes

Strain	*A. salmonicida* cell-free supernatant	*A. salmonicida* cell-free supernatant + biosurfactant
*C. violaceum*^a^	+	−
CV026^a^	+	−
*A. tumefaciens*^b^	+	−

a Purple pigmentation by violacein production.

b Blue pigmentation by β-galactosidase enzyme induction.

Since quorum sensing in *Vibrio anguillarum* is related to biofilm formation (Croxatto *et al*., 2002; Dobretsov *et al*., 2009), this strain was used to evaluate the effect of the biosurfactant on growth and biofilm formation. The results indicate that the concentration of biosurfactant required for maximal inhibition of biofilm formation was is 60 mg l^−1^ (Fig. 3C). No effect on *V. anguillarum* growth by the biosurfactant was detected (Supplementary Fig. S4). Therefore, the inhibition of biofilm formation was associated with an effect of the biosurfactant on viable growing cells.

### *Cobetia* sp. strain MM1IDA2H-1 and its biosurfactant inhibit the expression of *A. salmonicida* virulence genes

**Figure 4:**
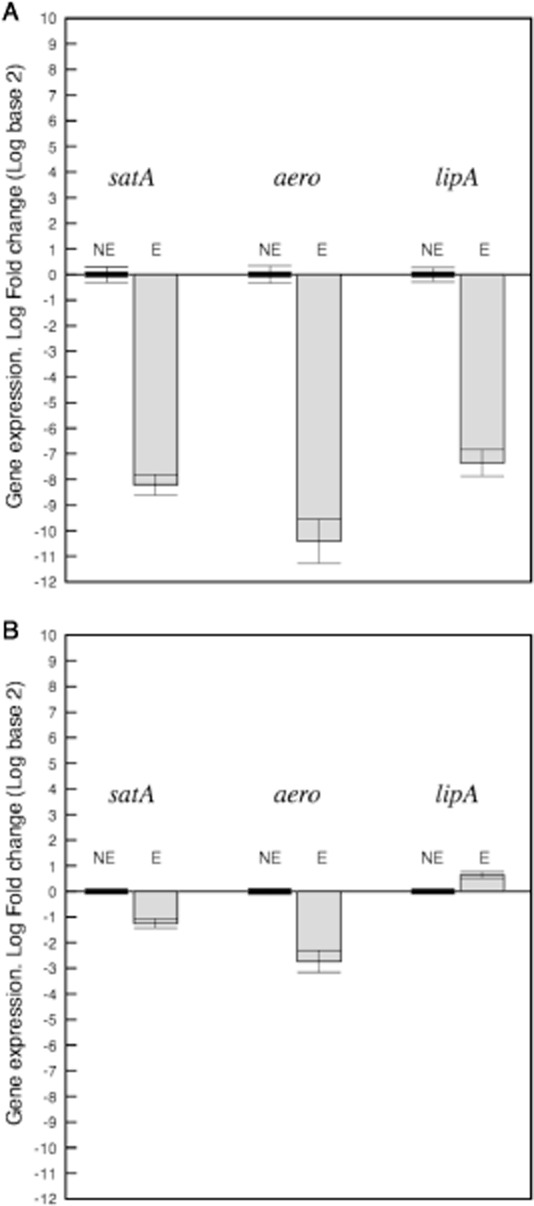
A. Expression of selected genes of virulence factors of *A. salmonicida* exposed to biosurfactant. Transcript levels of genes were measured by RT-PCR using RNA obtained from cultures (grown to an optical density at 600 nm = 1.0) of *A. salmonicida* exposed to 80 mg l^−1^ of biosurfactant (E). The control condition was the transcript levels of genes measured by RT-PCR using RNA obtained from cultures of *A. salmonicida* not exposed to biosurfactant (NE). In both, the experimental and control conditions, data were normalized to specific 16S RNA-gene of *A. salmonicida*. Data (bars are the standard deviation) are representative for three independent biological experiments.B. Expression of selected genes encoding virulence factors of *A. salmonicida* exposed to *Cobetia* sp. strain MM1IDA2H-1. Transcript levels of genes were measured by RT-PCR using RNA obtained from cultures of *A. salmonicida* grown with the *Cobetia* sp. MM1IDA2H-1 strain (optical density at 600 nm = 1.0) (E). The control condition was the transcript levels of genes measured by RT-PCR using RNA obtained from cultures of *A. salmonicida* (NE). Data from experimental and control conditions were normalized to specific 16S RNA genes of *A. salmonicida*. Data (bars are the standard deviation) are representative for three independent biological experiments.

The potential effect of *Cobetia* sp. strain MM1IDA2H-1 and its biosurfactant on the pathogenic behaviour of *A. salmonicida* was assessed by qRT-PCR analyses of the quorum-sensing-dependent virulence genes, glycerophospholipid cholesterol acyltransferase GCAT (*satA*) and aerolysin (*aero*). Additionally, the expression of the gene that encodes for lipase (*lipA*) was evaluated. When *A. salmonicida* was grown in the presence of the purified biosurfactant at a final concentration of 80 mg l^−1^, we observed a reduction in the expression of all the studied genes (Fig. 4A). The log (base 2) fold change reduction with respect to control condition (without the exposure to biosurfactant) was −8.21 for GCAT (*satA*); −10.41 for aerolisine (*aero*) and; −7.35 for lipase (*lipA*). No effect in transcript levels of the 16S-RNA gene of *A. salmonicida* was observed under any of the studied conditions. Notably, no effect was observed of the biosurfactant on *A. salmonicida* growth (Supplementary Fig. S5).

In the competition assay, when *A. salmonicida* was grown in the presence of *Cobetia* sp. strain MM1IDA2H-1, we observed a reduction in the expression of the GCAT (*satA*) and aerolysin (*aero*) transcripts with respect to the control condition (cells growing without competitor) (Fig. 4B). The fold change (log base 2) reduction was −1.2 and −2.73 for *satA* and *aero* respectively. Under the competition condition, the transcript levels for lipase (*lipA*) were not affected, with an increase of 0.65-fold with respect to the condition without a competitor. Using the 16S-RNA gene of *A. salmonicida* as a reference, no differences in growth were observed when this strain was grown with the *Cobetia* sp. strain MM1IDA2H-1.

## Discussion

In this work, a HDMB isolated from seawater samples was studied as a source of biosurfactants able to interfere in microbial interactions. The isolated strain, which uses DBT as the sole carbon and energy source, was identified as *Cobetia* sp. strain MM1IDA2H-1. It shares the highest sequence similarity to the strain *C. marina* DSM 4741. Despite this high degree of similarity, MM1IDA2H-1 showed phenotypic differences from the organism used as a reference in this study. This variation could be because *Cobetia* is a new genus with a growing number of described species (Romanenko *et al*., 2012) that exhibit wide geographical distribution with remarkable eco-physiological differences (Kim *et al*., 2009). Since the strain *Cobetia* sp. MM1IDA2H-1 is a moderate halophile with specific requirement of sodium to grow and able to use DBT as the sole carbon, we propose that this microorganism as a hydrocarbon-degrading bacterium indigenous to marine ecosystem.

At the quantities used, DBT broke down into particles in the liquid medium, generating two-phase systems (solid–liquid). This condition did not pose difficulties for microbial growth and can be explained by the early biosurfactant production with micelle formation, evidenced by a strong decrease in surface tension at the exponential growth phase. The rapid formation of micelles or vesicles may be necessary for the assimilation and metabolism of solid DBT since these lipid structures improve the bioavailability of solid substrates by promoting their dispersal and solubilization. The addition of solid substrates during fermentation significantly enhances microbial production of biosurfactants (Yeh *et al*., 2005). In this work, the production of lipid aggregates during bacterial growth in DBT was confirmed by TEM and EFM (Fig. 2B and D).

We observed that biosurfactant production in the hydrocarbon degrading marine strain also occurred in response to a biological stimulus, such as the presence of the potential competitor *A. salmonicida*. Although microbial competitors can induce biosurfactant production in marine bacteria (Dusane *et al*., 2011), this work is the first report in the case of HDMB. We postulate that the surface-active compounds in this microorganism play an important role in microbial fitness since they are involved in nutrient assimilation and displacement of potential competitors. Further studies should focus on establishing the mechanism by which the competitor induces biosurfactant production in *Cobetia* sp. MM1IDA2H-1. However, since in this work we used *A. salmonicida* as a competitor, we do not rule out the possibility that induction of biosurfactant production was mediated by quorum sensing.

Chemical analyses established that the biosurfactant was a mixture of fatty acids (C_n_ 3-OH). Fatty acids are components of cellular structures (membranes), but are also considered surface-active molecules when they diffuse outside the cell. When they reach their CMC, they form lipid aggregates (Satpute *et al*., 2010). Free fatty acids and aggregates are involved in microbial interactions. Certain fatty acids can act as diffusible messengers capable of disassembling the biofilm and interfering with the expression of virulence genes (Davies and Marques, 2009; Ryan *et al*., 2009). On the other hand, the presence of hydroxy fatty acids in the cell-free (filtered-centrifuged) supernatant may be related to outer membrane vesicles (OMVs). These lipid structures, which are produced and released by Gram-negative bacteria, can disseminate far from the cell to play biological roles on nutrient acquisition or cell-to-cell communication (Nevot *et al*., 2006; Kulp and Kuehn, 2010; Gutierrez *et al*., 2012). It has been reported that the OMVs participate in the intercellular trafficking of diffusible quorum-sensing signals (PQSs) through their packaging (Mashburn and Whiteley, 2005). In this context, it is reasonable to associate the biosurfactant produced by *Cobetia* sp. strain MM1IDA2H-1 with the inhibition of behaviours that depend on cell-to-cell communication. It is important to note that the maximal inhibition of quorum sensing (violacein phenotype and biofilm formation) was achieved when the biosurfactant was close to its CMC. This means that the inhibition effect occurs when surface-active monomers (in this case hydroxy fatty acids) spontaneously associate to form structures such as micelles, vesicles or lamellae, able to solubilize lipophilic molecules such as acyl-HSL signals. This is significant since in *V. anguillarum* and *A. salmonicida* biofilm formation and virulence depend on quorum-sensing circuits based on the membrane diffusible 3-oxo-C_n_-HSL (Croxatto *et al*., 2002; Rasch *et al*., 2007). In this work, when the *A. salmonicida* supernatant enriched in acyl-HSL was mixed with the biosurfactant at its CMC, the signal lost its ability to induce the quorum-sensing phenotype. This is explained by the physical properties of the AHLs signals and the biosurfactant ability to form lipid aggregates. However, we do not rule out that the biosurfactant through its free hydroxy fatty acids has a direct effect on quorum-sensing modulation.

Quorum sensing is an important cell-to-cell communication system responsible for cooperative behaviour in microbial interactions. This system of microbial interaction is based on diffusible molecules that act as molecular signals that allow microbial cells, in a sense, to be aware of each other and their environment and to coordinate their behaviour in response to stimuli, thus having a more efficient impact on their habitats as organized groups. Thus, microorganisms establish positive (symbiosis) or negative (pathogenic, competitive) intra-or interkingdoms relationships. Among the phenotypes controlled by quorum sensing are the production of virulence factors from a wide variety of bacterial pathogens of plants, animals and humans. Consequently, the inhibition of quorum sensing by interfering with or degrading chemical signals is a promising strategy to overcome the problem of microbial resistance to antibiotics. The repression observed in the expression of the *A. salmonicida* virulence genes, aerolysin (*aero*) and glycerophospholipid cholesterol acyltransferase (*satA*), is relevant since both genes are controlled by quorum sensing (Rasch *et al*., 2007). In comparison with the control conditions, the *aero* and *satA* genes were strongly repressed either when *A. salmonicida* was exposed to the purified biosurfactant or when it was grown in presence of *Cobetia* sp. strain MM1IDA2H-1. Interestingly, the assay using the biosurfactant also affected the *lipA* gene, which is not related to quorum sensing. Anyway it is clear that the use of the biosurfactant close to its CMC, in comparison with the assay with *Cobetia* cells, was more effective in controlling the pathogenic behaviour of *A. salmonicida*.

Hence, we propose that *Cobetia* sp. strain MM1IDA2H-1 produces hydroxy fatty acids that form structures when they reach their CMC that interfere with the lipophilic signals involved in cell-to-cell communication. The proposed mechanism can be understood as interference in quorum sensing by ‘signal hijacking’. The surface-active structures are produced to access nutrients and in response to the presence of potential competitors, improving the fitness of the *Cobetia* sp. strain MM1IDA2H-1.

## Experimental procedures

### Isolation and characterization of HDMB

The hydrocarbonoclastic marine bacterium was isolated from seawater samples collected from intertidal coastal ponds at Montemar, Chile. Enrichment cultures were prepared by inoculating the samples in 250 ml Erlenmeyer flasks containing 100 ml of Bushnell-Hass minimal medium (Difco, IL, USA) and 1% (w/v) of DBT (Merck, Darmstadt, Germany) as the only energy and carbon source. The flasks were incubated at 25°C for 21 days. Pure cultures were obtained by streaking the enrichment cultures on Marine Agar 2216 (Difco, IL, USA). Determination of cell morphology, Gram stain and the purity of colonies were made through microscopy using a single colony obtained from a culture grown in Marine Agar 2216 for 24 h. The isolate was identified by amplification, sequencing and analysis of 16S-RNA using the primers 27f and 1492r (Lane, 1991). The sodium requirement study was performed by inoculating a modified marine broth containing: tryptone 5 g l^−1^, yeast extract 1 g l^−1^ and 0.5%, 2.0%, 4.0, 6.0%, 8.0%, 10%, 12%, 14%, 16% and 20% (w/v) of sodium chloride (NaCl). Growth in the absence of sodium was determined by preparing marine broth with ultra-pure water. The growth at different temperatures was determined in Marine Broth 2216 (Difco, IL, USA) at 2°C, 6°C and 20°C and 30°C. The metabolic characterization was performed using the Biolog GN2 microplate system (Biolog, CA, USA) according to the manufacturer's instructions. The inoculated plates were incubated for 48 h at 30°C. The biochemical characterization was made through API NE strip system (Biomerieux, Marcy l'Etoile, France) following the manufacturer's instructions. Inoculated strips were incubated for 24–48 h at 30°C. For Biolog GN2 and API NE, the final concentration of NaCl in the inoculation fluid was corrected to 8% (w/v). The oxidase test was performed using Microbact Oxidase Strips (Oxoid, Adelaide, Australia). MICs to different antibiotics were determined in 96-well microtiter plates using the microdilution method using Luria–Bertani (LB) broth supplemented with NaCl (1% w/v). The following antibiotics were tested: amoxicillin, ampicillin, chloramphenicol, erythromycin, gentamicin, kanamycin, metronidazole, nalidixic acid, polymyxin B, rifampicin, stremptomycin and sulfonamide. Dilutions were prepared for each antibiotic. Each well was inoculated with 10^3^ cells from an early exponential-phase culture (OD_600_ = 0.4). Plates were incubated at 30°C for 24 h. MICs were established as the concentration of antibiotics where no growth was observed.

### Microscopic analysis

For microscopic studies, the *Cobetia* sp. strain MM1IDA2H-1 was cultured in Bushnell-Hass minimal medium (Difco, IL, USA) with 1% (w/v) of DBT (Merck, Darmstadt, Germany) or succinate 30 mM as the only carbon and energy source. For TEM, samples were taken from liquid cultures, filtered using 0.22 μm membranes (Millipore, Isopore) and processed for TEM using a Zeiss EM900 electron microscope. For EFM 1 ml of samples were extracted from cultures and stained with 10 μl of 3.34 μM SYTO 9 (Invitrogen, CA, USA) and BODIPY 505/515 (Invitrogen, CA, USA) at a final concentration of 25. The sample was incubated in the dark for 30 min. The green fluorescent SYTO 9 was used to stain nucleic acids visualizing bacterial cells in the sample. The treated samples were analysed by EFM with an Olympus IX81 epifluorescence microscope with a Chroma filter set (49011) and a UPlanSapo 60× objective.

### Metabolism of the aromatic hydrocarbon DBT

The metabolism involved in biodesulfurization was determined by amplifying and sequencing the *dszA*, gene with primers against conserved regions present in different bacterial isolates (Duarte *et al*., 2001). Conventional PCR amplification was performed using genomic DNA isolated with the QIAmp DNA extraction kit (QIAGEN, TX, USA). For amplification, the PCR mix contained 1 μl of DNA template, 5 μl of 10× reaction buffer, 2 μl of 2.5 mM dNTPs, 0.5 μl of 10 μM of each primer and 0.75 μl of 2.5 U of PfuUltra II Fusion HS DNA Polymerase (Agilent, CA, USA). The protocol for amplification was 30 cycles of 96°C for 1 s, 55°C for 10 s and 68°C for 30 s, with a final extension of 7 min at 68°C. The PCR products were visualized by 1.2% (w/v) agarose gel electrophoresis and purified using Qiaquick PCR Purification Kit (QIAGEN, TX, USA). Detection of degradation products from DBT was carried out on 0.22 μm membrane filtered supernatants of cultures grown on Busnell-Hass minimal media containing DBT as the sole carbon and energy source. The procedures for treatment, isolation and analysis of the metabolic intermediates from DBT degradation were performed according to a previous report (Oldfield *et al*., 1997). The organic phase was analysed by gas chromatography using a 178 Perkin-Elmer Autosystem XL equipped with a CPSIL-5CB capillary column.

### Obtaining inactivated cells of *A. salmonicida*

One hundred millilitres of late-exponential growth-phase cultures of *A. salmonicida* was filtered with a 0.22 μm membrane 1 l filtration unit (Nalgene, MF75). Filter devices were exposed to ultraviolet light at wavelengths of 240 nm under a UV lamp for 15 min. Cells from the filters were then freeze-dried at −82°C and 5 mtorr in a lyophilizer (Virtis, Benchtop K).

### Obtaining acyl homoserine lactone-enriched cell-free supernatant

One hundred millilitres of late-exponential growth-phase broth cultures of *A. salmonicida* or *C. violaceum* were respectively centrifuged at 4.000 *g* at 4°C for 30 min in a refrigerated centrifuge (Eppendorf, 5810R). The supernatants were filtered with a 0.22 μm membrane 1 l filtration unit (Nalgene, MF75) and stored in darkness at 4°C. The cell-free supernatants were used as source of acyl homoserine lactones as previously described (Rumbaugh, 2011).

### Production of biosurfactant during bacterial growth in response to DBT and microbial competitor

The presence of surface-active compounds produced by the isolated strain was established by the surface tension (ST) reduction of the culture medium using a DuNouy tensiometer (*CSC*, NOS 70535), and by the stability of the emulsion formed with hexadecane (Merck, Darmstadt, Germany). Erlenmeyer flasks containing 200 ml of Bushnell Hass with DBT were inoculated with 200 μl of a 16 h culture of the isolated strain grown at 30°C. The flasks were incubated at 30°C and 200 r.p.m. At different incubation times, 10 ml of sample was taken for ST measurement. The effect of potential competitors on biosurfactant production was determined by adding 0.1% (w/v) of inactivated *A. salmonicida* cells to Erlenmeyer flasks containing Bushnell-Hass minimal medium (Difco, IL, USA) with succinate 30 mM as the only carbon source, previously inoculated with the isolated marine hydrocarbonoclastic strain. Surface tension was measured at different times as described above. The emulsification index (EI_24_) was determined by adding 1 ml of the filtered supernatant on 2 ml of hexadecane, and vortexing for 2 min. EI_24_ was evaluated after 24 h according to previous description (Das *et al*., 2009). The critical micelle concentration (CMC) of the purified biosurfactant was estimated by plotting surface tension against log of biosurfactant concentration.

### Obtaining biosurfactant by fermentation

*Cobetia* sp. strain MM1IDA2H-1 was grown in 2 l of Bushnell-Hass minimal medium (Difco, IL, USA) with 1% (w/v) of DBT (Merck, Darmstadt, Germany) as the only carbon and energy source. The culture was made in a 3 l jar of a Biotron LiFlus-GX bioreactor at pH 7.2, 300 r.p.m., saturation of O_2_ and 30°C for 48 h. At the end of incubation, 2 l of culture was centrifuged at 13.000 *g* at 4°C for 30 min in a refrigerated centrifuge (Eppendorf, 5810R). Finally, the supernatant obtained was filtered through a 0.22 μm membrane in 1 l units (Nalgene, MF75).

### Chemical characterization

The filtered cell-free supernatant was treated with chloroform/methanol/water in a 2:2:1 ratio. After phase separation, chloroform was evaporated and the dried residue was quantified. After quantification, 1 ml BF_3_ (Boron Trifluoride) solution was added, and the sample was dried with nitrogen gas. The sample was placed in an oven at 40°C for 45 min. After cooling the sample, 2 ml of heptane and 4 ml of saturated NaCl solution were added and mixed by vortexing. After vortexing, the sample was centrifuged at 7.000 *g* for 10 min. For gas chromatographic (GC) analysis, 1 ml of the heptane phase was placed on a Hewlett-Packard 5890 series II gas chromatograph (Hewlett-Packard Company, USA) equipped with a split-splitless injector and a flame-ionization detector, and connected to a LDC Analytical D-2500 integrator for storing and data plotting. The mass spectral data were analysed by using the Wiley 138 library. The FTIR analysis was carried out in a Broker Vector 22-FT spectrometer, using potassium bromide (KBr) pills. The wave number was expressed as cm-1. The 1H, 13C (DEPT 135) spectra was recorded in CDCl3 solutions on a Bruker Avance 400 Digital NMR spectrometer, operating at 400.1 MHz for 1H and 100.6 MHz for 13C, and were referenced to the residual peaks of CHCl3 at δ 7.26 ppm and δ 77.0 ppm for 1H and 13C respectively.

### Interaction with cell-to-cell quorum sensing communication

*Chromobacterium violaceum* strains ATCC 12472 and CV026 (McClean *et al*., 1997) were used to determine the potential antagonism effect on quorum sensing signals by inhibiting pigmentation, as has been suggested (Rumbaugh, 2011). For the inhibition test, wells of microtiter plate containing 150 μl of LB broth were inoculated with 20 μl of a 16 h culture of *C. violaceum*. The biosurfactant was then added to the respective wells at final concentration of 5, 10, 50 and 100 mg l^−1^. The plates were incubated at 30°C for 24 h. Following incubation, the optical density of each well at 600 nm was measured on a microplate reader (Autobio Phomo Labtec Instruments) to evaluate the effect of the biosurfactant on growth.

### Interaction of the biosurfactant with AHLs of *A. salmonicida*

For signal biosurfactant interaction, wells of a microtiter plate filled with 100 μl of LB broth were inoculated with 10 μl from an overnight culture of strain CV026 and incubated at 30°C for 16 h. Then, the cell-free supernatant of *A. salmonicida* or *C. violaceum* were respectively mixed with the biosurfactant (final concentration of 100 mg l^−1^) by vortexing for 5 min and added to wells containing the strain CV026. For quorum sensing induction, the acyl homoserine lactone-enriched cell-free supernatant was added in wells containing the strain CV026. The plates were incubated for another 24 h at 30°C and analysed for violacein production. *Agrobacterium tumefaciens* NTL4(pZLR4) (Farrand *et al*., 1996) carrying a *traG::lacZ* reporter fusion was used for the detection of AHLs in *A. salmonicida* supernatants. This strain does not produce AHLs and contains the *traG::lacZ* reporter gene that is induced when its transcriptional activator TraR is cognated with an exogenous AHL. For testing, wells of microtiter plate filled with 150 μl of AB medium were inoculated with 20 μl of a 24 h culture of *A. tumefaciens* NTL4(pZLR4) and incubated at 30°C. Then, the cell-free supernatant of *A. salmonicida* was added to each well. The ability to induce quorum sensing was assessed by the induction of the β-galactosidase enzyme, measured colorimetrically (blue pigmentation) by adding X-gal (5 μl for each 10 ml of culture) from a stock solution (20 mg ml^−1^).

### Inhibition of biofilm formation

The effect of the biosurfactant produced by *Cobetia* sp. strain MM1IDA2H-1 on biofilm formation was evaluated with *Vibrio anguillarum*. The initiation of biofilm was measured by the crystal violet method (O'Toole and Kolter, 1998) using polyvinyl chloride 96-well plates containing: 2.0, 20, 40, 60 and 80 mg l^−1^ of the purified biosurfactant. Absorbance (at 540 nm) was measured on a microplate reader (Autobio Phomo Labtec Instruments). Previously, the effect of the biosurfactant on growth was studied by inoculating Erlenmeyer flasks containing 100 ml of Marine Broth 2216 medium supplemented with 80 mg l^−1^ of biosurfactant with 200 μl of an overnight *V. anguillarum* culture. The inoculated flasks were incubated at 20°C and 200 r.p.m. in an orbital incubator (Lab Companion, SK-300). Turbidity at 600 nm was recorded during the incubation.

### Effect on the expression of *A. salmonicida* virulence genes

*Aeromonas salmonicida* was grown on TSB exposed to a final biosurfactant concentration of 80 mg l^−1^ (E). An unexposed (NE) culture of *A. salmonicida* was used as a control condition. Cultures were incubated at 20°C and 200 r.p.m. until they reached the mid-exponential phase (OD_600_ ≈ 1.5). After incubation, the cells were separated from the culture by centrifugation at 4°C and 4000 *g* for 15 min. Cells were frozen at −80°C for RNA extraction. Previously, the effect of the biosurfactant on growth was studied by inoculation with 200 μl of an overnight *A. salmonicida* culture of Erlenmeyer flasks containing 100 ml of TSB medium supplemented with 80 mg l^−1^ of biosurfactant. The inoculated flasks were incubated at 20°C and 200 r.p.m. in an orbital incubator (Lab Companion, SK-300). Turbidity at 600 nm was recorded during incubation.

### Competition assay between *Cobetia* sp. strain MM1IDA2H-1 and *A. salmonicida*

For the competition assay, Erlenmeyer flasks containing 100 ml of TSB supplemented with 3% (w/v) of NaCl were inoculated with 200 μl of independent overnight cultures of the *Cobetia* sp. strain MM1IDA2H-1 (100 μl) and *A. salmonicida* (100 μl) (E). The turbidity of the overnight cultures at 600 nm was measured and adjusted with sterile TSB medium to obtain inoculums with the same cell ratio. Viability and inoculum sizes of the *Cobetia* sp. strain MM1IDA2H-1 and *A. salmonicida* were checked by streaking 100 μl on Marine Agar 2216 and TSB agar respectively. For the control condition (*A. salmonicida* not exposed to competitor), flasks containing 100 ml of TSB supplemented with NaCl (3% w/v) were inoculated only with *A. salmonicida* (OD_600_ ≈ 0.1) (NE). The inoculated flasks were incubated at 20°C and 200 r.p.m. in an orbital incubator (Lab Companion, SK-300). When an OD_600_ ≈ 1.5 was reached, cells were separated from the culture by centrifugation at 4°C and 4000 *g* for 15 min, and frozen at −80°C for RNA extraction.

### Relative quantifications (qRT-PCR) of *A. salmonicida* virulence factor genes

The mRNA levels of the following genes were quantified by qRT-PCR: lipase *(lipA)*, aerolysin *(aero)* and Glycerophospholipid cholesterol acyltransferase *(satA)*. RNA from *A. salmonicida* cells was extracted using TRI reagent (Ambion, CA, USA) and treated twice with RNAase-free DNAase (Promega, WI, USA) to eliminate traces of DNA. The resulting RNA was used as templates for reverse transcription and quantitative real-time PCR, using One-Step Brilliant III Ultra Fast QRT-PCR Master Mix (Stratagene, CA, USA), according to the manufacturer's instructions. Relative quantification of virulence factors was performed using the following primer sets (Nam and Joh, 2007): lipase *(lipA)*, aerolysin *(aero)*, Glycerophospholipid cholesterol acyltransferase *(satA)*, and the primers for a specific region of *A. salmonicida* 16S-RNA gene as normalizer. *Aeromonas salmonicida* not exposed to the biosurfactant or the *Cobetia* sp. strain *MM1IDA2H-1* cells was used as a calibrator for relative quantification. The transcript abundance of each RNA preparation was assayed in triplicate using an Mx3000P real-time PCR machine (Stratagene, CA, USA). Resulting cycle threshold values from qRT-PCR assays were analysed by the relative standard curve method and normalized to 16S-RNA gene expression. The changes in *lipA, aero* and *satA* transcript levels, relative to those in unexposed *A. salmonicida*, are given as means and standard errors of the means determined for at least three RNA preparations for each experimental condition, each of which were assayed in triplicate.

### Strains and culture conditions

*Cobetia marina* ATCC 25374 was grown in Marine Broth 2216 at 30°C and 200 r.p.m. The *Cobetia* sp. strain MM1IDA2H-1 was grown in Bushnell-Hass minimal medium (Difco, IL, USA) supplemented with 1% (w/v) of DBT (Merck, Darmstadt, Germany) or 30 mM succinate at 30°C and 200 r.p.m. For biochemical characterizations *Cobetia* sp. strain MM1IDA2H-1 was grown in Marine Broth 2216 at 30°C and 200 r.p.m. *Aeromonas salmonicida* subsp. *salmonicida* ATCC 33658 was grown in Tryptic Soy Broth (TSB) (Oxoid, Adelaide, Australia) at 20°C and 200 r.p.m. *Vibrio anguillarum* ATCC 19264 was grown in marine broth 2216 at 20°C. *Chromobacterium violaceum* ATCC 12472 and *C. violaceum* strain CV026 were grown on LB broth (USB Corporation, OH, USACleveland) at 30°C and 200 r.p.m. *Agrobacterium tumefaciens* NTL4(pZLR4) (Farrand *et al*., 1996) was cultured at 30°C in AB minimal medium supplemented with gentamicin (150 μg ml^−1^).

### Nucleotide sequence and accession numbers

The DNA sequences were analysed using the blast search algorithm to estimate similarity to other 16S-RNA gene sequences obtained from the NCBI/GenBank (http://www.ncbi.nlm.nih.gov/BLAST/).

## Supporting information

Table S1 Characteristics of *Cobetia* sp. strain MM1IDA2H-1 compared with the reference strain *Cobetia* marina DMS 4741.

Table S2 MICs of selected antibiotics.

Fig S1 ^1^H-NMR spectra obtained in CDCl3 solutions on a Bruker Avance 400 Digital NMR spectrometer of biosurfactant produced by *Cobetia* sp. strain MM1IDA2H-1, treated with Chloroform/Metanol/water in a 2:2:1 ratio.

Fig S2 13C-NMR (DEPT 135) spectra obtained in CDCl3 solutions on a Bruker Avance 400 Digital NMR spectrometer of biosurfactant produced by *Cobetia* sp. strain MM1IDA2H-1, treated with Chloroform/Metanol/water in a 2:2:1 ratio.

Fig S3 Comparison the mass spectrums of Wiley 138 library with the GC-MS chromatograms of biosurfactant produced by *Cobetia* sp. strain MM1IDA2H-1, treated with Chloroform/Metanol/water in a 2:2:1 ratio, obtained in a Hewlett-Packard 5890 series II gas chromatograph.

Fig S4 Growth of *Vibrio anguillarum* exposed and unexposed to the biosurfactant from *Cobetia* sp. strain MM1IDA2H-1, measured by optical density at a way length of 600 nm. *Vibrio anguillarum* was grown in marine broth supplemented with 80 mg l-1 of the biosurfactant.

Fig S5 Growth of *Aeromonas salmonicida* exposed and unexposed to the biosurfactant from *Cobetia* sp. strain MM1IDA2H-1, measured by optical density at a way length of 600 nm. *Aeromonas salmonicida* was grown in Trypotone Soy broth supplemented with 80 mg l-1 of the biosurfactant. 405 C. Ibacache-Quiroga et al.
