# Supplementary material for: The hydrocarbon-degrading marine bacterium Cobetia sp. strain MM1IDA2H-1 produces a biosurfactant that interferes with quorum sensing of fish pathogens by signal hijacking
Source: Microb Biotechnol. 2013 Jan 2;6(4):394–405. doi: 10.1111/1751-7915.12016 (PMC3917474; doi:10.1111/1751-7915.12016)
Supplement: Table S1 — Characteristics of Cobetia sp. strain MM1IDA2H-1 compared with the reference strain Cobetia marina DMS 4741. [file mbt20006-0394-sd1.doc]

| **Table 1.** Characteristic of *Cobetia* sp. strain MM1IDA2H-1 | | |
| --- | --- | --- |
|  | *Cobetia sp. strain* MM1IDA2H-1 | *Cobetia marina* DSM4741 |
| *Cell morphology* | straight rods | straight rods |
| *NaCl growth range* | 1.0 % - 18 % | 0 -18 % |
| *Growth at 0 % of NaCl* | - | + |
|  |  |  |
| *Enzymatic activity* |  |  |
| Oxidase | + | - |
| Nitrate reduction | - | - |
| Beta-galactosidase | + | + |
| Lysine decarboxylase | - | - |
| Ornitine decarboxylase | - | - |
| Indol production | - | - |
| Arginine Dihydrolase | - | - |
| Urease | - | - |
| Beta-glucosidase | - | - |
|  |  |  |
| *Metabolism of Dibenzothiophene* |  |  |
| Apparition of hydroxybiphenyl | + | ND |
| Detection of *dszA* gene | + | ND |
|  |  |  |
| *Metabolism of:* |  |  |
| alfa-Cyclodextrin | - | - |
| Tween 40 | + | + |
| Tween 80 | + | + |
| L-Arabinose | - | - |
| D-Arabitol | - | + |
| D-Cellobiose | - | - |
| i-Erythritol | - | - |
| D-Fructose | + | + |
| l-Fucose | - | - |
| D-Galactose | + | + |
| Gentobiose | - | - |
| alfa-D-Glucose | + | + |
| m-Inositol | + | + |
| Sucrose | + | + |
| D-Threalosa | + | + |
| Turanosa | + | + |
| Xylitol | + | - |
| Pyruvic Acid Methyl Ester | + | + |
| Succinic Acid Mono Methyl Ester | + | + |
| Acetic Acid | - | - |
| Cis-Aconitic Acid | + | + |
| Citric Acid | + | + |
| Formic Acid | - | + |
| D-Gluconic Acid | + | + |
| D-Glucosaminic Acid | + | - |
| D-Glucoronic Acid | + | - |
| alfa-Hydroxybutyric Acid | - | - |
| beta-Hydroxybutyric Acid | + | + |
| gamma- hydroxybutyric Acid | + | + |
| p-Hydroxy Phenylacetic Acid | - | - |
| alfa Keto Butyric Acid | - | - |
| alfa Keto Glutaryc Acid | + | + |
| alfa Keto Valeric Acid | - | - |
| D,L- Lactic Acid | + | + |
| Succinic Acid | + | + |
| Bromosuccinic Acid | + | + |
| Succinaminic Acid | + | + |
| Glucoronamide | - | - |
| L- Alaninamide | - | - |
| D-Alanine | + | + |
| L-Alanine | + | + |
| L-Alanyl-glycine | + | + |
| L-Aspargine | + | + |
| L-Aspartic Acid | + | + |
| L-Glutamic Acid | + | + |
| Glycyl-L-Aspartic Acid | - | - |
| Glycyl-L- Glutamic Acid | + | + |
| L-Histidine | - | - |
| Hydroxy-L-Proline | - | - |
| L-Phenylalanine | - | - |
| L-Proline | + | + |
| L-Pyroglutamic Acid | + | + |
| D-Serine | - | - |
| L-Serine | + | + |
| L-Threonine | - | - |
| D,L- Carnitine | + | + |
| gamma- Amino Butyric Acid | + | + |
| Urocanic Acid | - | - |
| Inosine | + | + |
| Uridine | + | + |
| Thymidine | - | - |
| Phenylethylamine | - | - |
| Putrescine | - | - |
| 2-Aminoethanol | - | - |
| 2,3-Butanediol | - | - |
| Glycerol | + | + |
| D,L-alfa-Glycerol Phosphate | + | + |
| alfa-D-Glucosa-1-Phosphate | - | - |
| D-Glucose-6 Phosphate | - | - |
