# Supplementary material for: The hydrocarbon-degrading marine bacterium Cobetia sp. strain MM1IDA2H-1 produces a biosurfactant that interferes with quorum sensing of fish pathogens by signal hijacking
Source: Microb Biotechnol. 2013 Jan 2;6(4):394–405. doi: 10.1111/1751-7915.12016 (PMC3917474; doi:10.1111/1751-7915.12016)
Supplement: Table S2 — MICs of selected antibiotics. [file mbt20006-0394-sd2.doc]

| **Supplementary Table 2.**  Minimal Inhibitory Concentrations (MICs) of selected antibiotics | |
| --- | --- |
| Antibiotic | MIC (mg l^-1^) |
| Amoxicillin | 0.25 |
| Ampicillin | 0.125 |
| Chloramphenicol | 8 |
| Erythromycin | 128 |
| Gentamicin | 8 |
| Kanamycin | 8 |
| Metronidazole | >256 |
| Nalidixic acid | 32 |
| Polymyxin B | 128 |
| Rifampicin | 64 |
| Streptomycin | 8 |
| Sulphonamide | >128 |
