# Supplementary figures and images for: The hydrocarbon-degrading marine bacterium Cobetia sp. strain MM1IDA2H-1 produces a biosurfactant that interferes with quorum sensing of fish pathogens by signal hijacking
Source: Microb Biotechnol. 2013 Jan 2;6(4):394–405. doi: 10.1111/1751-7915.12016 (PMC3917474; doi:10.1111/1751-7915.12016)

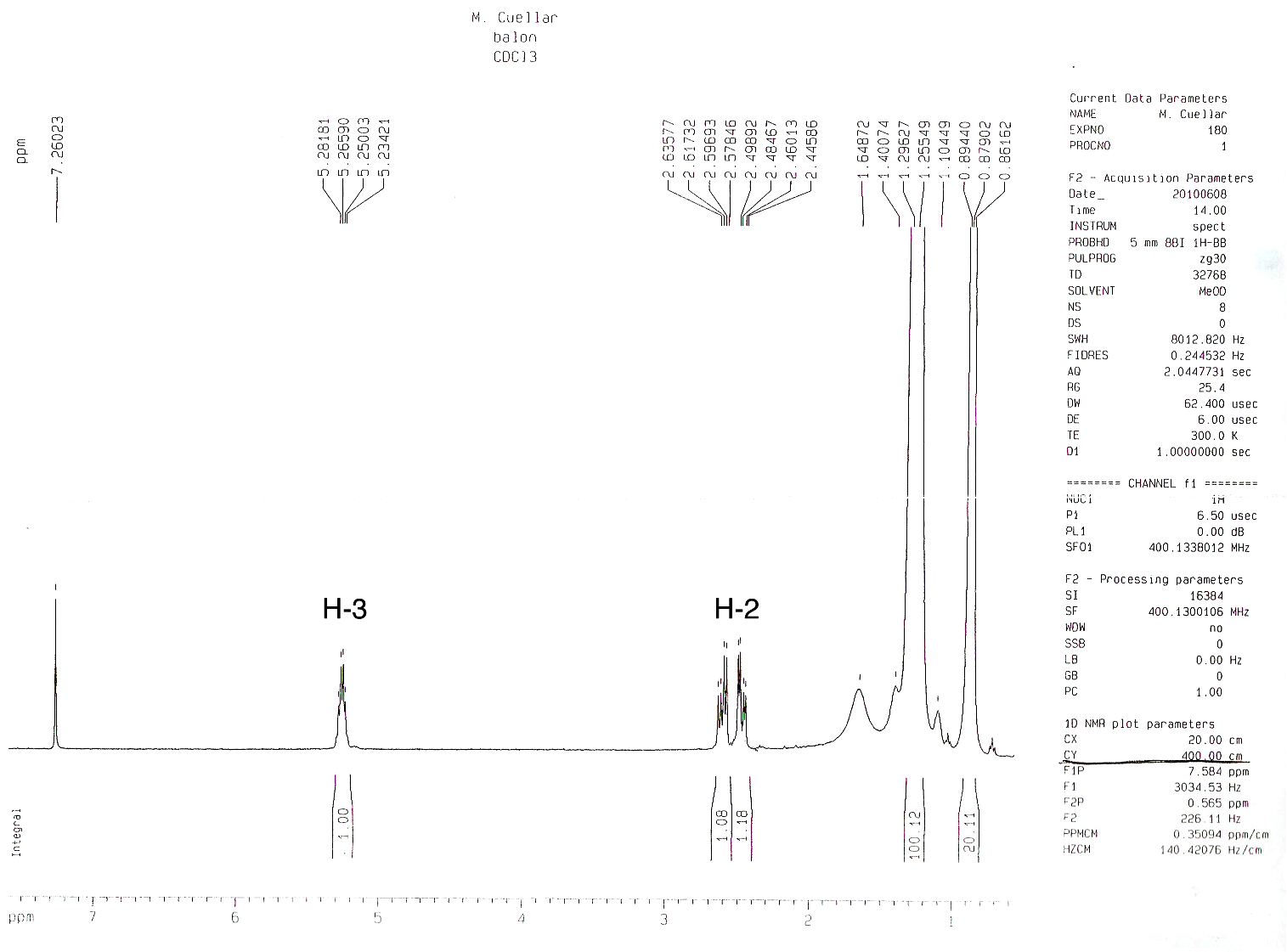

Supplement: Fig S1 — 1H-NMR spectra obtained in CDCl3 solutions on a Bruker Avance 400 Digital NMR spectrometer of biosurfactant produced by Cobetia sp. strain MM1IDA2H-1, treated with Chloroform/Metanol/water in a 2:2:1 ratio. [file mbt20006-0394-sd3.tiff]

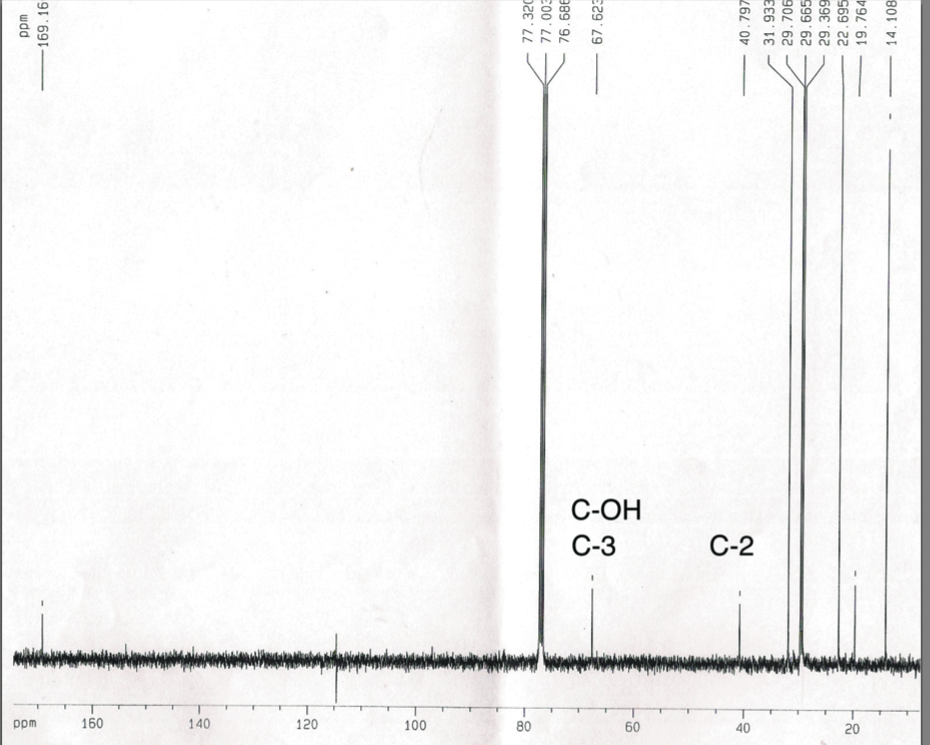

Supplement: Fig S2 — 13C-NMR (DEPT 135) spectra obtained in CDCl3 solutions on a Bruker Avance 400 Digital NMR spectrometer of biosurfactant produced by Cobetia sp. strain MM1IDA2H-1, treated with Chloroform/Metanol/water in a 2:2:1 ratio. [file mbt20006-0394-sd4.tiff]

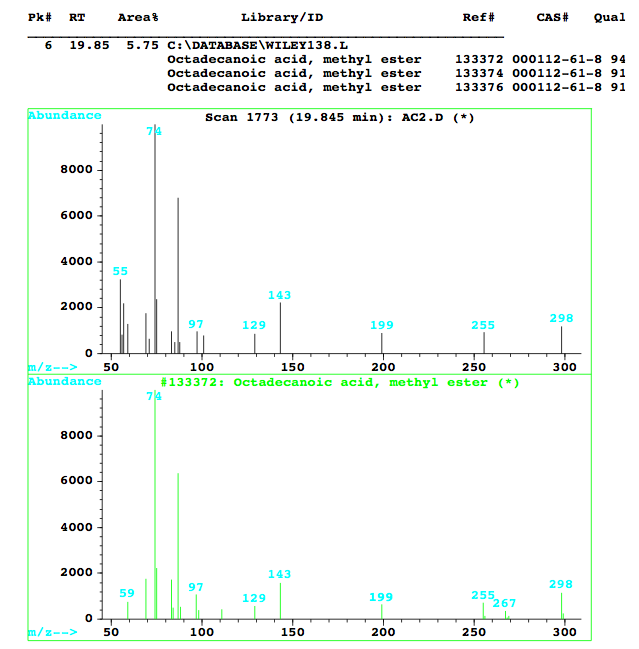

Supplement: Fig S3 — Comparison the mass spectrums of Wiley 138 library with the GC-MS chromatograms of biosurfactant produced by Cobetia sp. strain MM1IDA2H-1, treated with Chloroform/Metanol/water in a 2:2:1 ratio, obtained in a Hewlett-Packard 5890 series II gas chromatograph. [file mbt20006-0394-sd5.tiff]
